# The End of the Beginning? Temporality and Bioagency in Pandemic Research

**DOI:** 10.1093/jhmas/jrae006

**Published:** 2024-05-03

**Authors:** Mandisa Mbali

**Affiliations:** University of Cape Town, South Africa

**Keywords:** AIDS in South Africa, intellectual history, transdisciplinarity, temporality, bioagency, biocapital

## Abstract

This paper deals with the ways in which the intellectual and political history of AIDS can assist in the chronological conceptualization of a pandemic such as COVID-19 as it is unfolding. It problematizes the idea of pandemic “beginnings” and “ends” to show that such definitions are shaped by the disciplinary location and thematic foci of relevant scholars. Central to this analysis is the notion that ethical and political contexts affect research on a pandemic in different ways at national and global levels at various points in its trajectory. The article develops this argument in relation to two main themes: firstly, with reference to the history of AIDS research in South Africa; secondly, with the philosophical concept of bioagency to understand the ways in which viruses and humans co-shape the course of epidemics over time. I first make the case for the development of historically informed, long-term ethnographic studies of COVID-19. Using bioagency as a point of departure to consider viruses as social actors, the essay then critiques the notion of bioinformationalism as catalyzing the widening accessibility of biomedical research. Instead, I discuss the biotechnology and pharmaceutical industries as protagonists in the operation of biocapital. I argue that the history of AIDS in South Africa can provide methodological and theoretical insights into how to interpret an unfolding epidemic, outlining an ambitious transdisciplinary research agenda for thinking about the temporality of a pandemic spanning the different, interconnected, scales of life.

## Introduction

As we approach the end of the third year of the COVID-19 pandemic, are we at the end of the beginning or the beginning of the end of the outbreak? As regards the beginning of the end of COVID-19, it is a strong hypothesis based on available evidence that this is far from the case. In particular, there are many unanswered questions about the epidemiological and biomedical evidence, especially with regard to evolving viral diversity and uncertainty about the physiological implications of long COVID. Sociologically speaking, it may be the case that we are at the end of the beginning. Whole populations have now lived with lockdowns and travel quarantines. A great many people have had COVID-19 themselves, know people who have the disease, have lost their lives, or have had someone they know lose their lives. Every country has experienced the reality, fears, and responses to the health-systems and economic ramifications of the pandemic.

The discovery of vaccines effective in warding off serious clinical manifestations of COVID-19 and related deaths have been tested and received regulatory authority. However, international vaccine access inequity means that while relatively large numbers of people in the Global North have now been vaccinated against COVID-19, many in developing countries continue to lack vaccine access. If the invention of effective vaccines such as those developed by BioNTech/Pfizer and Moderna may mark the end of the beginning of the pandemic, we need to consider some of the powerful social forces hindering, at a minimum, the beginning of its end, which may conceivably be significantly shaped by the universal global roll-out of vaccines.

Historians of medicine have often been approached by media organizations to offer insights on how we can use past pandemics to understand COVID-19 as a social phenomenon.^1^ COVID-19 has also provided an opportunity for historians to reflect further on the intellectual history of public health in relation to epidemics, especially how new research methodologies were implemented in different parts of the world over time. It is in this context that I use this essay to discuss two problems facing historians of medicine since the late twentieth century who may wish to re-conceptualize epidemics not only in biomedical terms but also, simultaneously, in terms of public health politics and research in the philosophy of science. I argue for a historically informed transdisciplinary approach designed to translate COVID-19 research into policy and practice: research which should be aimed at hastening the beginning of its end. First, I examine the relative influence of societal stigma and modes of transmission on the trajectories of early AIDS and COVID-19 social science research. The history of AIDS in South Africa shows, I argue, the importance of long-term, in-depth historically informed ethnographic research into the social drivers of and barriers to access to medicines.

Second, I make the case that historians can effectively employ the concept of bioagency to understand the complex intersections between viruses and humans which may dynamically co-shape the course of epidemics over time. The concept of bioagency is especially useful in informing early efforts to periodize the history of COVID-19. In particular, I argue that we need to examine the *social* implications of the unprecedented generation and sharing of very large amounts of biological information pertaining to genetic sequencing of SARS-CoV-2, its immunological impact, and related vaccines’ efficacy. The causal relationships between changing natural and social worlds have not, however, been unidirectional. Instead, as histories of prior epidemics have shown, the spread of infectious diseases has also been driven by social and cultural phenomena. Reiterating this can help us avoid a sort of biotriumphalism where ending future epidemics merely involves scientific technofixes such as genetic sequencing and vaccine development.

The question of the extent to which diseases are socially constructed or biologically determined is a well-established debate in the history of medicine. In 1992, Charles Rosenberg presented a compelling model of how the biology of disease “framed” social understandings of it: disease was “a structuring factor in social situations…a social actor and mediator.”^2^ Rosenberg described the relationship between disease and society as “an interactive system” where “formal understandings of disease entities interact with their manifestations in the lives of particular men and women.”^3^ The interfaces described were mainly between “patient and physician,” between “physician and family,” and “between medical institutions and medical practitioners.”^4^

While Rosenberg’s model of understanding the social construction of diseases remains persuasive, when writing histories of AIDS and COVID-19 the rapidly evolving fields of virology and immunology have reshaped the historical trajectories of both diseases. In recent decades, the genetic evolution of these viruses and how they act on cells have been mapped by scientists in finer detail with ever greater volumes of data in real time. The medical breakthroughs which have resulted from these scientific developments have determined the social course of these pandemics.

The concept of bioagency is a useful way to reflect on these scientific developments since the 1980s. The deployment of bioagency in the history of medicine should not, however, be triumphalist in its understandings of social advancements occasioned by biomedicine. While bioinformaticians, geneticists, and virologists may have rendered unprecedented volumes of scientific information on SARS-Cov-2 accessible, biotech and pharmaceutical companies (or biocapital) have constrained access to biomedical knowledge and related clinical interventions in the form of long, exclusive patents.^5^ In general, I describe what a historical methodological and theoretical approach can offer to scholars in diverse disciplines who wish to study the politics of an unfolding pandemic. Even more ambitiously, I suggest a way in we may also be able to use the concept of bioagency to imagine altering the politics of an unfolding pandemic, thereby shaping our collective human co-evolution with SARS-CoV-2 over time.

## Theorizing Bioagency

Philosophers of biology such as Joshua Charles Skewes and Cliff Hooker have developed the concept of bioagency in response to the fact that older accounts of freedom and reason do not account for causality in

complex dynamical systems that that involve simultaneous interactions under multiple feedback. In them causal event-threads have neither beginning nor ending and so offer no resolution of causal responsibility. These problems are especially acute for those systems involving *self-organised process architectures and globally organised feedback loops as living organisms do*.^6^

When we think about the biological world, there are two sets of processes: those that are endogenous and metabolic in nature and those that are external and environmental. These are constantly in interaction with each other.^7^ One key element of bioagency is “self-directed anticipatory learning” which can be related to the fact that an organism needs to “match its capacities to its opportunities and risks moment-to-moment so as to continually satisfy its autonomy.”^8^ Central to “the autonomous bio-agent model” are the ideas that living things are dynamic and can autonomously self-regulate and interact with environmental processes.^9^ Pointing to the dynamism of “bio-organisational integration,” Skewes and Hooker argue that “reason and freedom” are “interlocked and developing capacities…expressed in the dual capacities of intentionality and intelligence.”^10^

If the concept of bioagency applies to living things, we should first consider whether and how a virus lives. Here we can note that there is a debate within biology about whether the metabolically parasitic nature of viruses renders them “non-alive.”^11^ The first position is that viruses are alive because there are viruses and virophages which are parasites of other viruses. Contrary to this view is the position that they are not alive because they cannot reproduce without a cellular host. Instead, Eugene Koonin and Petro Starokadomskyy have proposed what they term a “replicator paradigm” whereby all entities which belong to the realm of biology have the ability to replicate.^12^ They dismiss the question of whether viruses live by arguing that “the question is effectively without substance because the answer depends entirely on the definition of life or the state of ‘being alive’ that is bound to be arbitrary.”^13^ Indeed, as they mention, host-parasite co-evolution is a fundamental part of evolution.^14^ The view of a virus as non-life has important ramifications: as Warwick Anderson has pointed out, simply considering a virus as an alien disease through the “mechanical model of contact and contamination” can lead to militarised and xenophobic responses to such diseases.^15^

Soraya de Chadarevian and Roberta Raffaetà have proposed that we use the term “intra-action” instead of “interaction” to show that two forms of agencies – virus and humans – “are not distinguished before their encounter but arise from it.”^16^ They draw upon the work of Karen Barad who has proposed “agential realism” as a new philosophical approach to thinking about the relationships among “matter, discourse and causality.”^17^ Barad is both a social theorist and a quantum physicist and as such her work has chiefly drawn upon Niels Bohr’s insight that when measuring subatomic particles some properties can be determined only to the specific exclusion of others: in other words, “the experimental apparatus” adopted shape what we measure to the exclusion of other properties.^18^ In emphasising the entanglements of matter and meaning and agency she has theoretically pushed historians of medicine, anthropologists, and those in Science and Technology Studies (STS) far beyond typical binaries between social constructionism and realism.^19^ An outstanding task, however, remains producing the narrative social history of a virus using these this philosophical approach of agential realism; provisional early histories of COVID-19 may present a way forward as I suggest in the fourth part of this paper.

As “humans are the main vectors for [SARS-CoV-2] proliferation,” this means that the virus is “always and already both natural and human made.”^20^ Finally, Chadarevian and Raffaetà make the case for us to “take into consideration the dynamic interactions of humans and other species in specific historical settings.” To think about “how to live humanely in a pandemic,” we could consider the social life of humans and its enmeshment in the viral world such as to enable us to produce “an anthropology of the virus.”^21^

Bioagency can offer a dynamic and complex approach to understanding the biological and social determinants of diseases. How we view diseases may be a question of the webs and scales of life which interact in changing ways over time: a virus such as SARS-CoV-2 enters and acts on cells in our bodies in directed ways aiming at matching opportunities (spread via aerosol transmission) in terms of how we interact via talking or breathing in closed spaces at close proximity or travelling. This can be contrasted with a disease such as AIDS, where HIV enters and acts on our bodies in directed ways (via the exchange of bodily fluids) aimed at matching opportunities in terms of how we exchange them (via unprotected sexual intercourse, use of injecting drugs with the sharing of needles, or use of contaminated blood products). This has shaped the histories of both epidemics in fundamentally different ways. Societies have, of course, responded differently to each: while opportunities for SARS-CoV-2 to be exchanged have been reduced via use of masks, social distancing and quarantines, and restrictions in movement, AIDS prevention efforts have been dominated by programs and policies aimed at moderating sexual behavior. These social measures in turn have shaped SARS-CoV-2’s ability to replicate and, hence, its evolutionary success.

There are, therefore, “simultaneous interactions” between the viruses and humans which are fundamental to shaping the trajectory of each epidemic, including COVID-19.^22^ SARS-CoV-2’s ability to spread and mutate depends on a dynamic feedback mechanism between our bodies and the way we organize our social lives; its trajectory will be heavily shaped by the virus itself as a biosocial entity. Even if we concede that viruses may only be life-like and possessing of much lesser agential capacities (as a much less complicated or determining type of biological entity), we must consider their interaction with humans’ physiology and anatomy.

## “Slow Research” in an Unfolding Pandemic: The Case of AIDS in South Africa

Pessimistically (or perhaps realistically), in late 2023 we may only be at the “end of the beginning” of the COVID-19 pandemic. From early 2022, broader media started to call COVID-19 “endemic”; however, as Jacob Steere-Williams has pointed out, it is important to recognize that there are “cultural and political and not always scientific reasons for labelling a disease endemic.”^23^ He has gone on to argue that since the mid-twentieth century, Western conceptions of endemicity have allowed global health policy actors to marginalize older infectious diseases as public health problems merely affecting countries in the Global South.^24^

In the early COVID-19 pandemic era, what are some of the lessons in terms of how public health donor agendas and the stigma related to a disease can shape public health research to alter its course? As I have argued, problematic discourses in relation to the “end of AIDS,” technofixes and celebrity scientists and activists have been emphasized both in the “AIDS world” and in popular discourse.^25^ This has been to the detriment of the funding and development of effective societal interventions aimed at tackling the social drivers of persistently high rates of preventable HIV infections and deaths. The discovery of COVID-19 vaccines is of analogous import in terms of the pandemic’s trajectory to that in relation to AIDS occasioned by the discovery of effective combination anti-retroviral therapy in so much as both these interventions have prevented the needless loss of life. The global public good of the universal adoption of such interventions is beyond dispute. However, the reasons why people may not accept them need to be understood with reference to social and cultural factors which require long-term ethnographic research.

I would like to also propose the persuasiveness of Vincanne Adams, Nancy Burke, and Ian Whitmarsh’s call for a movement of “slow research” in global health.^26^ Critiquing the dominance of the singular conceptions of “fast research,” where specific and targeted interventions are deemed to be universally replicable, they have called for longer-term ethnographic research. Slow research recognizes the importance of local actors and regional cultural differences, promoting approaches which have been effective over longer periods of time. This agenda also adopts exploratory or open-ended approaches which do not rigidly anticipate the answers to questions by means of pre-determined hypotheses; it also encompasses an “iterative process” where research is more “reciprocal” and “responsive to context.”^27^ Adams and others have also expressed skepticism in relation to rapid appraisal research aimed at generating data which flattens out the complexity of social phenomena requiring longer term examination.

The approach they suggest is, however, not lacking in nuance: they are not in opposition to fast research proposing targeted public health and biomedical interventions which work in multiple contexts. Instead, their view is that it can be augmented by longer-term ethnographic research. Their analysis of the intellectual terrain of global health is also guided by a sophisticated understanding of the political economy behind the dominant design of research interventions which are driven by what they term “a research industry that demands globally comparable metrics” which do not necessary reflect local realities.^28^ They have also emphasized the importance of resisting “market logics” where technological interventions are framed as “investments” which can be “profitable.”^29^ In a contemporary context where public health researchers’ fame as produced through minutes-long TED talks and YouTube lectures is often valourized, they note that: “The amount of data as information produced by the speed and scale of new technologies and markets is astounding; however the commitments to this form of information production risks effacing the iterative back and forth process as well as the sustained depth of familiarity which comes with longer and often messier engagements.”^30^

The intellectual history of AIDS research in South Africa is a useful reminder of the importance of long-term medical anthropology in public health research. This is far from true only in relation to South Africa. Discussing the importance of long-term community-based public health research (including projects dealing with AIDS) in Kenya, Charles Salmen and colleagues have argued that “alongside the gold standard, multi-site, cluster randomised control trials we [in global public health] celebrate, let us pay respect to the inventive, nuanced, and locally-specific inquiries that grow out of deep community-based engagement.”^31^ There is a need for slow research dealing with the social and cultural context in which COVID-19 is occurring and this is especially true in an African context. This will take some time and it may not be able to directly and adequately inform public health interventions at this early phase, but it must be undertaken in earnest.

Comparatively speaking, AIDS and COVID-19 show that both infectious diseases’ modes of transmission and societal stigma can shape the different trajectories of social science research. For COVID-19, as a virulent, airborne infectious disease, in-person COVID-19 research carries several risks, especially among the unvaccinated. There were examples of xenophobia and homophobia revealed in the early phase of the COVID-19 pandemic, among other things in relation to its emergence in China and association with homophobia in South Korea.^32^ It could, however, be argued that owing to the large-scale society-wide rapid airborne transmission of COVID-19, there was a lesser degree of stigma in comparison with a similarly early period in the early AIDS pandemic.

For AIDS, from relatively early on in its history, scientists knew that it was sexually transmitted via bodily fluids (with patterns of transmission mimicking Hepatitis B). In the case of social science AIDS research, on the face of it, the risk of HIV transmission in conducting oral history interviews or engaging in ethnographic research interviews was practically non-existent.^33^ However, as several scholars have demonstrated, the sexual mode of HIV transmission and its early association with gay men, sex workers, and people who used injectable narcotics meant that there was a great deal of stigma and discrimination associated with those living with the disease, which shaped AIDS research priorities and interests. This can be seen in the way in which AIDS research was conducted in South Africa.

As Carla Tsampiras has shown, virologists published early articles in the *South African Medical Journal* (*SAMJ*) which made problematic assertions exclusively associating early AIDS cases with White gay men, foreclosing the reality of cross-racial sexual transmission between men. Here she significantly notes that, “The differences between ‘African’ and ‘Western’ AIDS were reinforced long before science was able to differentiate between HIV clades and speculate as to their geographical origin.”^34^ Moreover, in the first article in the *SAMJ* dealing with AIDS in prostitutes, they were bracketed as being members of a “high risk group” based not on South Africa data, but upon an early study on HTLV-III prevalence among prostitutes and their clients in central Africa.^35^ This can also be interpreted as linking African AIDS to a racist essentialized notion of African promiscuity.^36^ Finally, different clades of the virus came to be associated with particular races and sexual orientations: and so, Clade B cases were linked to gay men (all of whom were assumed to be White) and Clade C infections were associated with heterosexuals.^37^

In her sophisticated historicization of virological and epidemiological AIDS research in South Africa, Tsampiras moves us away from a biotriumphalist account of science. Conversely, while attentive to the scientists’ early racist, sexist and homophobic social constructions of AIDS as a disease, she does not, for example, dispute viral diversity or HIV’s mechanism of action in terms of transmission. Bioagency can help us to avoid the binary of either social constructionist or scientific realist accounts of disease in ways which chime well with Tsampiras’s rich scientific history.

AIDS entered South Africa in a period defined by racial segregation justified in terms of Calvinist Christianity. Sodomy was illegal and the infamous “three men at a party” clause forbade more than two men to meet for “homosexual” gatherings.^38^ While there were early efforts by gay organizations to educate gay men about the threat posed by AIDS, the landscape of gay organizing was heavily divided along the lines of race.^39^ In as much as gay sexual activity, organizations, the use of dildos (including for condom promotion), and the propagation of frank gay-appropriate and empowering sex education were illegal, it was ethically and practically difficult to recruit key informants to thoroughly qualitatively study AIDS among gay men in the 1980s.^40^ Indeed, the periodization of the production of a myriad of rich legal, anthropological, and gender-focused studies of AIDS and gay men demonstrates that key publications were produced during a transition period in the 1990s, and thus many years after the disease’s identification, such as the signature queer studies volume by Cameron and Gevisser published in 1995.^41^

Early anti-apartheid social science AIDS research and activism in South Africa were also hindered by the broader politics of race, gender and sexuality in the period. During the apartheid era, the stigmatization of, and efforts to control, Black sexuality were key planks of government repression on the grounds of race and gender. In particular, the state feared that the Black population would swamp the White population and so implemented an elaborate and coercive family planning program which targeted Black South African women (including forced and coerced and Depo Provera injections).

When heterosexual AIDS emerged among truck drivers, migrant laborers, and sex workers in the 1980s, events such as the Chamber of Mines’s repatriation of 13,000 Malawian miners between 1988-1992 did little to foster openness about living with HIV.^42^ It is in this context that early social science studies were not always well received in progressive political circles. This was clear, for example, when researchers at the Sociology of Work Programme (SWOP) at the University of the Witwatersrand brought their study highlighting the risk of HIV transmission among mine workers in 1988-1989 to the attention of the National Union of Mineworkers (NUM). The union initially interpreted their findings as racist and so the study did not substantially influence their policies and programs.^43^

The SWOP study focused squarely on the issue of how the unjust migrant labor system was linked to sex work in ways which were influenced by women’s economic marginalization. However, the NUM’s rejection of this study needs to be situated with reference to earlier colonial medical and public health assumptions about syphilis in Africa. Indeed in 1991, Randall Packard and Paul Epstein argued that anthropologists’ initial narrow focus on how the spread of AIDS was driven by Africans’ sexual behavior mirrored earlier colonial rhetoric of syphilis as being spread by problematic, urbanized “detribalised” natives.^44^ These early anthropological approaches to AIDS were also divorced from an understanding of their living conditions and the social determinants of health (including, for example, the migrant labor system). This narrowing of research foci led to data which was highly open to methodological critique.^45^

Early South African epidemiological studies pointed to the early role of prostitutes in the spread of HIV, patterns of migrants having higher rates of infection and women becoming more susceptible to HIV at much earlier age compared to men.^46^ However, these studies did not fully explain *why* young women were over-represented in terms of those living with HIV or the social nature of the links between HIV infection and migrancy. Even as AIDS was peaking as the leading cause of death in South Africa in 2001, many studies looking at social and cultural aspects of the epidemic lacked longitudinal depth and often strictly dealt with contemporaneous phenomena related to the epidemic. For example, in 2002 Delius and Walker argued that longer term social science studies were needed to properly understand the nature of AIDS in South Africa:

The enormous urgency of the issues in tandem with the funding cycles and priorities of donor organisations have tended to promote fairly rapid and sometimes formulaic forms of…[social science] research. This work has yielded important insights but…the complexity of the issues require[s] in-depth and long-term historical and sociological research. This substantive research should also inform and respond to rigorous and large-scale quantitative research.^47^

By the early twenty-first century, some years after the disease’s emergence, carefully designed, long-term ethnographic studies of the social drivers of HIV infections began to be emerge, which responded to quantitative research yet did not necessarily inform it. For example, Catherine Campbell’s ethnography *Letting Them Die* provided an in-depth account of what motivated migrant mineworkers and sex workers to have sex with multiple partners without using condoms: the mineworkers felt it was more manly and also already faced a great deal of danger in the course of their work and so were fatalistic in relation to unprotected sex.^48^

Mark Hunter’s *Love in the Time of AIDS* showed that sex work was far from the only way in which the feminization of poverty rendered women vulnerable to HIV infection.^49^ Through a long-term ethnographic study in Mandeni township and informal settlement, he found that poorer, younger women were having sex with multiple, older, wealthier men in exchange for *both* cash *and* desirable commodities. Hunter argued that these relationships needed to be placed in historical context: high levels of long-term unemployment meant that men could no longer afford to form their own households and so they exchanged cash and goods as signs of love and affection to form relationships.

Yet it appears that little of this humanities and social science research was directly integrated into health sciences, public health, or applied behavioral sciences. For instance, as late as 2009, Vasu Reddy, Theo Sandfort, and Letitia Rispel noted that despite the fact that there was already ample research on homosexuality in law and the humanities disciplines, there was an “absence of homosexuality in the health sciences, public health and behavioral sciences literature about AIDS in South Africa.”^50^ They argued that disparity in focus could be attributed to the inherently conservative influence of funding for large behavioral sciences research projects and the epidemiology of the AIDS epidemic in South Africa, which is predominant heterosexual.^51^

The findings of these long-term ethnographic studies on barriers to preventing new HIV infections have yet to be fully integrated into policy and practice in South Africa. For example, there is inadequate provision of LGBTIQ-friendly sexual and reproductive health care in the country. In many government facilities, homophobic and transphobic stigma and discrimination present significant barriers to LGBTIQ people accessing quality HIV treatment and prevention (including Pre-Exposure Prophylaxis) programs.^52^ Similarly, sex work remains criminalized and this hinders the development of HIV prevention and treatment services appropriate to their needs.^53^ Finally, the feminization of poverty has yet to be adequately addressed in South Africa: this, in turn, drives young women’s engagement in transactional sex with multiple concurrent partners.^54^

In the intellectual history of AIDS in South Africa, there is however a more promising example of how slow research can document, and thereby promote, effective activist-driven community-based public education in encouraging the wider usage of a new medical intervention. This activism was necessary in a context where the country’s president denied the scientific facts in relation to AIDS, with devastating consequences for people living with the illness. From 1999, just as South Africa’s HIV infection rates were placing AIDS as the leading cause of death in the country, President Thabo Mbeki espoused views which his critics branded as “AIDS denialism.” In particular, he questioned whether HIV was the cause of AIDS, the diagnostic validity of HIV tests, and the safety and efficacy of combination anti-retroviral (ARV) therapy.

A year before he espoused these views, a new social movement called the Treatment Action Campaign (TAC) had been formed to demand universal access to anti-retroviral drugs to prevent mother-to-child-transmission (PMTCT) and for use for chronic treatment of HIV. Given the lack of public understanding of the science behind AIDS, one of the first tasks facing the movement was to build public awareness about anti-retrovirals (ARVs). A particularly challenging element of this was the fact that the drugs were relatively new and in the case of combination therapy, drug resistance meant the regimens changed over time. Once again here, bioagency can be called upon to understand the enmeshment between biological and social elements of a pandemic. This is because the activists had to explain how HIV operated at a molecular level, including the dynamism of its viral diversity. Differently put, the activists explained scientific research about HIV’s evolution and actions at a small scale to influence the society-wide course of a pandemic.

As was documented in great detail by several historically informed, ethnographic studies, they used at least five different effective methods to counteract the AIDS denialism of the president. As visual anthropologists showed, they encouraged women living with HIV to produce artistic images showing how they understood HIV in relation to their bodies: a method known as “body mapping.”^55^ Historian Rebecca Hodes has written about how film-maker-activists produced documentaries and popular television shows explaining the science behind HIV and ARVs.^56^ I have also documented HIV treatment activists’ effective use of various facets of everyday township life including the use of choirs, sports matches, and church services to educate communities about the safety and efficacy of ARVs.^57^ The history of in-depth historical and anthropological research into mistrust of science and popular efforts to counter it indicates the fruitfulness and importance of taking a longer and culturally enmeshed view of the societal phenomena which hinder or advance widespread adoption of a new medical intervention responding to a new pandemic disease.

This indicates that in all contexts there needs to be a systematic chronological mapping (enumeration and comparison) of in-depth, long-term, qualitative humanities research into COVID-19 vaccine hesitancy and denial and what motivates people to both get vaccinated themselves and to convince others to do so. There should also be an analysis of the funding which is being made available by public health donors for in-depth, long-term, qualitative humanities’ studies of COVID-19 in societies. It is reasonable to hypothesize that it may be inadequate and that more projects of this nature are required. I would also argue that the need for such research is especially acute in African contexts.

## Theorizing “The End”? Bioagency, Biocapital and Health Activism

Conceiving of the end of COVID-19 is impossible in the absence of universal access to vaccines to reduce the severity of illness and the probability of death in infected patients. At the time of writing, biocapital continues to deploy long and inflexible pharmaceutical patents, which hinders the manufacture of cheaper versions of COVID-19 vaccines. Since the late 1990s, AIDS activists in South Africa and their global allies in the international HIV treatment access movement have challenged such patents for essential medicines, including ARVs. Many of the same organizations and networks which formed this movement are also heavily involved in contemporary vaccine access activism, such as Universities Allied for Essential Medicine (UAEM), Médecins Sans Frontières (MSF), and the Treatment Action Campaign (TAC). As historians start to consider writing about the COVID-19 pandemic (and a host of other ongoing epidemics), we may wish to conceptualize this political struggle between biotechnology companies and activists in the Global South over access to vaccines against the theoretical backdrop of bioagency. In the case of COVID-19, political will and scientific endeavor have been spurred on by the combination of exceptional virulence of the virus and the very high incidence rate. Moreover, the extent of data-sharing and the rapidity of vaccine development have fundamentally altered the medical threat posed by the Sars-CoV-2 virus. However, shortly after the vaccines’ development, social actors began to hinder the epidemiological amplification of the benefits of this biomedical breakthrough to Global North countries.

Bioagency (considering how the viral world shapes human experience and vice-versa) may be in clear evidence in global health law and policy. When we look at global vaccine inequity, a key issue is the need to challenge biotech and pharmaceutical companies’ long, inflexible patents on COVID-19 medical products and to ensure that more affordable copies of the vaccine can be made available in developing countries. In his book *Biocapital: The Constitution of Postgenomic Life*, Kaushaik Sunder Rajan argues that “the life sciences represent a new face, and a new phase, of capitalism.”^58^ Biotech companies are most often funded by venture capitalists and they often become listed companies whose share price is determined by speculation on stock markets (notably investors on Wall Street).

Furthermore, we should challenge accounts that imply that COVID-19 has substantially advanced the realization of the right to science (scientific knowledge). Instead, the issue of corporate and institutional gate-keeping in pharmaceutical patenting is crucial to understanding the uneven and jagged endings for the first stage of this pandemic. While it is laudable that scientists are sharing their research so openly and at such a rapid rate via data sharing, pre-prints, and rapid peer review, the research on vaccines must go from bench (laboratories) to bedside (clinics and vaccination sites). The vaccines are “biologic” drugs (also described as “living medicines”) and cheaper versions made through different mechanisms of action are called “biosimilars.” As South African health activists argued in relation to the breast cancer drug trastuzumab, when patents expire on biologic drugs and biosimilars are produced and licenced, access to them substantially widens as they become more affordable.^59^

Inversely, in discussions on the trajectory of COVID-19, a repeated narrative has been the celebration of the open sharing of research data. For example, Petar Jandrić has argued that we must understand SARS-Cov-2 genetic sequencing with reference to bioinformationalism: here he is talking about multiple different things.^60^ Firstly, in the context of the overwhelming march of digitization of knowledge since the late twentieth-century, biology took a major digital turn. So, for example, when the human genome was sequenced the “analogue world of biology” was translated into the “digital world of the computer.”^61^ Furthermore, Jandrić goes on to argue that,

In our postdigital age, contagious diseases such as Covid-19 are at the same time biological (they arrive from nature and affect human bodies), social and cultural (they elicit socially and culturally constructed responses) and digital (Covid-19 research is enabled and powered by digital technology). Developed within a postdigital context, [the] world’s response to the threat of Covid-19 says a lot about the viral nature of our modernity.^62^

The sharing of scientific research into COVID-19 can also be analyzed through a bioinformationalist methodology.^63^ From early on in the COVID-19 pandemic, peer-reviewed research publications became open access, research findings were made available via pre-print servers, and researchers shared “interim and final research data relating to the outbreak.”^64^ However, it was made clear to authors that the sharing of data and pre-prints did not pre-empt publication in these journals. Springer’s *Nature* made its SARS-Cov-2 and COVID-19 research available for free. This new approach to rapid information-sharing was unprecedented and “within weeks” Chinese scientists “had sequenced the viral genome, deciphering the virus’s genetic code.”^65^ Crowe has also claimed that “when the story of the coronavirus (2019-n-COV) is finally written, it might well become a template for the utopian dream of open science – where research data is shared freely, unrestrained by competition, paywalls and patents.”^66^ According to this framework, the scientific world of COVID-19 has been flattened through open access and viral sharing of research.

In late 2021, the unintended consequences of such sharing of genomic research were in full evidence in the case of scientists at the Network for Genomic Surveillance’s detection of the Omicron variant in Botswana and South Africa. This genomic data had been rapidly shared with scientists around the world through the Sanger institute’s SARS-CoV-2 sequencing database.^67^ Of greatest concern to the South African scientists was that this this variant had a huge number of mutations to the spike protein enabling the infection of human cells. South Africa’s government and the team of scientists announced that a new variant of SAR-CoV-2 had been identified in southern Africa, which the WHO declared to be a new variant of concern. The diplomatic results were swift: many high-income countries immediately banned travel to southern African countries, with severe socio-economic consequences, especially for the tourism sector.

As Keymantri Moodley and colleagues have argued, this did not represent global health ethics in terms of the sharing of the benefits and burdens of the rapid distribution of scientific knowledge in relation to SARS-CoV-2.^68^ While genomic data has been gathered and shared by southern African researchers, patients in these countries have often been the last to benefit from updated Omicron-tailored vaccines which were themselves derived from “the detection and early sharing of [genomic] information about variants of concern.”^69^

This exclusion of Africans from enjoying the fruits of scientific progress is not without precedent. Since the late 1990s, South African AIDS activists and their international allies in the movement for universal access to essential medicines have been arguing that long, inflexible patents should not be a barrier to patients’ access to life-saving medical technologies. In the late 1990s, the country was in the midst of an AIDS epidemic where the disease was becoming its leading cause of death. In turn, ARVs which could effectively prevent perinatal transmission of the disease and AIDS-related deaths in HIV-positive patients remained prohibitively expensive. From 1997-2000, forty multinational pharmaceutical companies tried to block the Mandela administration from passing the Medicines and Related Substances Amendment Act into law. This act was aimed at enabling the issuing of compulsory licencing where patents could be broken to enable the production of more affordable generics.

The Durban International AIDS conference of 2000 marked a signal founding moment in the global access to medicines movement. Following street protests, direct action, and a media-savvy campaign against the litigation, the pharmaceutical companies dropped their case. American AIDS activists then turned to successfully pressing for the Clinton administration to revoke its threats to place South Africa on the Special 301 trade watch list, which would have effectively introduced trade sanctions. Activists from the movement then set their sights on demanding changes to the WTO’s Trade and Related Intellectual Property (TRIPS) agreement.^70^ Following its Doha declaration on the TRIPS Agreement and Public Health, countries facing global health emergencies were able to use compulsory licensing to enable production of generic medicines. This agreement resulted in fifty-two such compulsory licences between 2001-2007 and facilitated a radical growth in access to generic ARVs, fundamentally altering the course of the global AIDS pandemic.^71^

COVAX, or the vaccines-related facility of the Access to COVID-19 Tools (ACT) Accelerator, has largely failed to enable a timely, effective Africa-wide roll-out of COVID-19 vaccines. Founded in April 2020 by GAVI (the Vaccines Alliance), CEPI (the Coalition for Epidemic Preparedness), and the WHO, it is aimed at advancing fundraising, the negotiation of bulk purchase, and the globally fair distribution of COVID-19 vaccines. It is worthy of mention in the context of the need for patents to be broken to ensure universal access to the vaccines; put differently, it illustrates the shortcomings of approaches to the equitable global distribution of pharmaceutical products based upon what civil society critics such as MSF have referred to as “charity” alone.^72^

Despite the laudable global health solidarity-related goals of the facility, vaccine nationalism came to dominate the worldwide distribution of the vaccines. Governments in the Global North refrained from accessing vaccines through COVAX and, instead, opted to engage in bilateral negotiations with patent-holding pharmaceutical manufacturers, thereby obtaining the vast majority of the world’s vaccine supply.^73^ Then, when faced with the Delta variant of the virus, India prohibited the export of vaccines developed by AstraZeneca and produced by the Serum Institute of India. When vaccines were delivered later than planned, vaccinated patients missed their second doses. Moreover, some of the doses delivered were close too close to expiry to be distributed at health facilities in a timely fashion. Finally, “COVAX’s timid approach to issues such as sharing vaccine technology…infuriated advocates…[for the] lifting of IP rights” to enable a radical increase in their manufacture.^74^ These delay missteps and delays in the roll-out of vaccines in countries in the Global South had been linked to vaccine hesitancy, indicating the long shadow of the initial failures of COVAX.^75^

By contrast, on the international stage, both India and South Africa have argued for a patent waiver for COVID-19 vaccines (since October 2020) to expand manufacturing capabilities, especially in the Global South. More than 100 low-income countries supported the proposal. One remarkable turn of events was that on 5 May 2021, US President Joe Biden announced support for a patent waiver; however, the UK, Canada, Japan and Australia have expressed opposition to the patent waiver. World Trade Organization (WTO) Director-General Ngozi Okonjo-Iweala has been strongly in support of the proposal, and on 9 June 2021 talks began; however, these talks have been moving at a glacial pace.

## Conclusion

At the current conjuncture, where effective vaccines (including booster shots) have been made widely available in countries in the Global North for some time, it is enticing to hypothesize that the world may have entered a phase in the life of the COVID-19 pandemic which may be termed the end of the beginning. Yet the relationships between viruses and humans are never disentangled and unidirectional. Here, the concept of bioagency can assist us in thinking through the complex and dynamic webs of connection between humans and the natural world, including viruses. At various levels, humans continue to coevolve with viruses, including that which causes COVID-19; however, this reality cannot be conflated with simplistic notions that “we need to learn to live with it [the SARS-CoV-2 virus].”

Given the dramatically positive results of COVID-19 vaccines it may seem that, from an immediate perspective, the trajectory of the pandemic is being primarily shaped by advances in biomedical research. There is certainly sense of data deluge among scientists, as they share an absolutely enormous amount of mostly digital information. The incredible speed, volume and sharing of – in some, important cases – critical biomedical findings can conceptually and politically overshadow the potential explanatory power, and public health applications, of studies describing people’s historically shaped, meaning-infused, lived experiences with COVID-19. Yet, from a broader perspective, we are reminded that if strides are truly to be made in ending the pandemic, humanities and social science research that articulates the longer-term contexts and trajectories of disease and medical intervention are also necessary. The history of AIDS is a reminder of the ways in which research into an epidemic shifted over the course of its outbreak and the explanatory power of long-term ethnographic research to understand the social dimensions of an unfolding pandemic.

It is illuminating to compare the trajectories of social research on AIDS to that on COVID-19 with the former being potentially informative on how to study the social drivers of the spread of a pandemic disease and the efficacy of popular education efforts to encourage the uptake of a new (and sometimes mistrusted) medical intervention to combat it. Here, a case can be made that scholars in the medical humanities and social sciences should not necessarily be the last in the line when it comes to research funding and agenda-setting to advance the universal, global roll-out of COVID-19 vaccines. Similarly, the relationships and dialogue among biomedical, epidemiological and medical humanities should not be one-sided: there should be ongoing two way sharing of research between scholars in the humanities and social sciences on the one side and epidemiologists, physicians and those in the biomedical sciences on the other. In order to imagine the beginning of the end of COVID-19, a vital element of which would be a universal global vaccine roll-out, biomedical and public health, humanities and social science, and global health legal studies aimed at advancing that goal must *coevolve together*.

At the same time, advancing a universal, global COVID-19 vaccine roll-out does not merely belong in the province of researchers (from whichever relevant discipline based on the question being posed). International relations and law can help us understand how long inflexible patents on essential medicines and the TRIPS agreement shape global access to essential medicines (including the vaccines). The concept of biocapital can prove useful in thinking through the role of biotechnology and pharmaceutical companies in blocking access to biologics such as the COVID-19 vaccine. Understanding the factors hindering access to affordable COVID-19 vaccines in developing countries is not merely a descriptive and technical exercise: at a deeper level, debates about access to biologic drugs must address the global ethical implications of life as capital at a fundamental human level.

Perhaps, the different ways we think about the ever-changing web of COVID-19 as disease and pandemic may be partly shaped by where and how we focus on the scales of life. While biomedicine offers us various views of how viruses get and work under our skin what historians of medicine can, and should, provide are societal accounts of how viruses become us over time.

## Footnotes

1 For illustrative purposes see, Gina Kolata, “How Pandemics End,” *New York Times*, 10 May 2020; Laura Spinney, “Closed Borders and ‘Black Weddings’: What the 1918 Flu Teaches Us About Coronavirus,” *The Guardian*, 11 March 2020. Kolata cites a wide array of historians of medicine offering insights from prior epidemics, including Jeremy Greene, Allan Brandt, Dora Vargha, Mary Fissell, Frank Snowden, David Jones, and Naomi Rogers. Similarly, the Spinney article cites Howard Phillips. I have also been interviewed on provisional reflections on how the COVID-19 pandemic will affect history. Mandisa Mbali, “PODCAST|Covid’s Milestone Moments: How the Pandemic Will Affect History: Opinion Kwezi Science Reports,” interview by Tanya Farber, *Sunday Times*, 9 November 2021, https://new.timeslive.co.za/articles/premium/opinion-and-analysis/2021-11-09-podcast--covids-milestone-moments-how-the-pandemic-will-affect-history.

2 Charles E. Rosenberg, “Framing Disease: Illness, Society and History,” in *Framing Disease: Studies in Cultural History*, ed. Charles E. Rosenberg and Janet Golden (New Brunswick, NJ: Rutgers University Press, 1992), xviii.

3 Ibid., xxiii.

4 Ibid.

5 I unpack bioinformationalism later in this article with reference to Petar Jandrić’s work. Petar Jandrić, “Bioinformationalism: Information Theory and Genomics,” in Michael A. Peters, Petar Jandrić, Peter McLaren, “Viral modernity? Epidemics, Infodemics, and the ‘Bioinformational’ Paradigm,” *Educational Philosophy and Theory* 54 (2022): 675-697.

6 J.C. Skewes and C.A. Hooker, “Bio-Agency and the Problem of Action,” *Biology and Philosophy* 24 (2009): 283-300, 285, emphasis added.

7 Ibid.

8 Ibid., 289.

9 Ibid., 297-298.

10 Ibid., 294.

11 Koonin and Starokadomskyy offer an excellent analysis of this debate and propose a very interesting approach. Eugene V. Koonin and Petro Starokadomskyy, “Are Viruses Alive? The Replicator Paradigm Sheds Decisive Light on an Old but Misguided Question,” *Studies in History and Philosophy of Biological and Biomedical Sciences* 59 (2016): 125-134.

12 Ibid.

13 Ibid.

14 Ibid.

15 Warwick Anderson, “Not on the Beach, or Death in Bondi,” *Somatosphere*, 7 May 2020, http://somatosphere.net/2020/not-on-the-beach-or-death-in-bondi.html.

16 Soraya de Chadarevian and Roberta Raffaetà, “COVID-19: Rethinking the Nature of Viruses,” *History and Philosophy of the Life Sciences* 43 (2021): 2.

17 Karen Barad, *Meeting the Universe Halfway: Quantum Physics and the Entanglement of Matter and Meaning* (Durham: Duke University Press, 2007), 26.

18 Ibid., 19.

19 See for example, Chadarevian and Raffaetà, “COVID-19”; Kenneth R. Fleishmann, “The Evolution of Agency: the Spectra of Bioagency and Cyberagency,” *Information Society* 23 (2007): 361-371; Gregory Hollin, Isla Forsyth, Eva Giraud, and Tracey Potts, “(Dis)entangling Barad,” *Social Studies of Science* 47 (2017): 918-941.

20 Chadarevian and Raffaetà, “COVID-19.”

21 Ibid.

22 Here I am applying Skewes and Hooker’s concept of “simultaneous” interactions to Sars-CoV-2. See Skewes and Hooker, “Bio-Agency,” 285.

23 Jacob Steere-Williams, “Endemic Fatalism and Why it Will Not Resolve COVID-19,” *Public Health* 206 (2022): 30.

24 Ibid.

25 Mandisa Mbali, “African Achievers, Structural Barriers and the ‘End of AIDS,’” in *Forward, Upward, Onward? Narratives of Achievement in African and Afroeuropean Contexts*, ed. Ulrike Pirker, Katja Hericks, and Mandisa Mbali (Düsseldorf: Heinrich Heine University Books, 2020), 79-87.

26 Vincanne Adams, Nancy J. Burke, and Ian Whitmarsh, “Slow Research: Thoughts for a Movement in Global Health,” *Medical Anthropology* 33 (2014): 179-197.

27 Ibid., 192.

28 Ibid., 181.

29 Ibid., 186.

30 Ibid., 191.

31 Charles R. Salmen, Richard Magerenge, Louisa Ndunyu, and Shailendra Prasad, “Rethinking our Rigor Mortis: Creating Space for More Adaptive and Inclusive Truth-Seeking in Community-Based Global Health Research in Kenya,” *Global Public Health* 17 (2022): 4002-4013.

32 As regards homophobia in South Korea, see for instance, John P. DiMoia, “Contact Tracing and COVID-19: The South Korean Context for Public Health Enforcement,” *East Asian Science, Technology and Society: An International Journal* 14 (2020): 1-10; Sora Lee and Ryan Wong, “COVID-19 Responses of South Korea as Hybrids of Governance Modes,” *Frontiers in Public Health* 9 (2021): 654945. In relation to anti-Asian xenophobia, see for instance, Tyler T. Reny and Matt A. Barreto, “Xenophobia in the Time of Pandemic: Othering, Anti-Asian Attitudes, and COVID-19,” *Politics, Groups, and Identities* 10 (2022): 209-232; Yulia E. Chuvileva, Andrea Rissing, and Hilary B. King, “From Wet Markets to Wal-Marts: Tracing Alimentary Xenophobia in the Time of COVID-19,” *Social Anthropology* 28 (2020).

33 However, people living with HIV who publicly disclosed that they were living with the virus faced the risk of HIV-related hate crimes. See Mandisa Mbali, *South African AIDS Activism and Global Health Politics* (New York: Palgrave Macmillan, 2013).

34 Carla Tsampiras, “From ‘Dark Country’ to ‘Dark Continent’: AIDS, ‘Race,’ and Medical Research in the South African Medical Journal, 1980–1995,” *Journal of Southern African Studies* 41 (2015): 773-196.

35 Carla Tsampiras, “Removed from Context and Complexity: Women in Early AIDS Research in the South African Medical Journal, 1980-1995,” *Journal of Southern African Studies* 45 (2019): 128.

36 Tsampiras, “From ‘Dark Country’ to ‘Dark Continent,’” 786.

37 Mandisa Mbali, “The New Struggle: A History of AIDS Activism in South Africa, 1982-2003” (PhD diss., University of Oxford, 2009).

38 Mark Gevisser, “A Different Fight for Freedom: A History of South African Lesbian and Gay Organisation from the 1950s to the 1990s,” in *Defiant Desire: Gay and Lesbian Lives in South Africa*, ed. Mark Gevisser and Edwin Cameron (London: Routledge, 1995), 14-88.

39 Mbali, *South African AIDS Activism and Global Health Politics*.

40 Isaacs and McKendrick aptly described the psychological effects of homophobia and its implications for gay men’s experiences of AIDS in the period. Gordan Isaacs and Brian McKendrick, *Male Homosexuality in South Africa: Identity Formation, Culture, and Crisis* (Oxford: Oxford University Press, 1992).

41 Gevisser and Cameron, ed., *Defiant Desire*.

42 Wiseman Chijere Chirwa, “Aliens and AIDS in Southern Africa: The Malawi–South Africa Debate,” *African Affairs* 97 (1998): 53-79; Louis Grundlingh, “Government Responses to HIV/AIDS in South Africa as Reported in the Media, 1983–1994,” *South African Historical Journal* 45 (2001): 124-153.

43 The study I am referencing here is: K. Jochelson, M. Mothibeli, and J.P. Leger, “Human Immunodeficiency Virus and Migrant Labor in South Africa,” *International Journal of Health Services* 21 (1991): 157-173. This study was not significantly taken up by the union for reasons discussed. See Mbali, “‘The New Struggle.’”

44 Randall M. Packard and Paul Epstein, “Epidemiologists, Social Scientists, and the Structure of Medical Research on AIDS in Africa,” *Social Science and Medicine* 33 (1991): 771-794.

45 Ibid.

46 Q. Abdool Karim, S.S. Abdool Karim, B. Singh, R. Short, and S. Ngxongo, “Seroprevalence of HIV Infection in Rural South Africa,” *AIDS* 6 (1992): 1535-1539; Q. Abdool Karim, S.S. Karim, K. Soldan, and M. Zondi, “Reducing the Risk of HIV Infection among South African Sex Workers: Socioeconomic and Gender Barriers,” *American Journal of Public Health* 85 (1995): 1521-1525.

47 Peter Delius and Liz Walker, “AIDS in Context,” *African Studies* 61 (2002): 11.

48 Catherine Campbell, *Letting Them Die: Why HIV/AIDS Intervention Programmes Fail* (Bloomington: Indiana University Press, 2003).

49 Mark Hunter, *Love in the Time of AIDS: Inequality, Gender and Rights in South Africa* (Bloomington: Indiana University Press, 2010).

50 Vasu Reddy, Theo Sandfort, and Letitia Rispel, “Introduction,” in *From Social Silence to Social Science: Same Sex Sexuality, Gender and HIV/AIDS in South Africa*, ed. Vasu Reddy, Theo Sandfort, and Letitia Rispel (Cape Town: HSRC Press, 2009), xi-1.

51 Ibid., xiv.

52 Alex Müller, “Scrambling for Access: Availability, Accessibility, Acceptability and Quality of Healthcare for Lesbian, Gay, Bisexual and Transgender People in South Africa,” *BMC International Health and Human Rights* 17 (2017): 16; Diantha Pillay, Kayla Stankevitz, Michele Lanham, Kathleen Ridgeway, Mercy Murire, Elmari Briedenhann, Sarah Jenkins, Hasina Subedar, Theresa Hoke, and Saiqa Mullick, “Factors Influencing Uptake, Continuation, and Discontinuation of Oral PrEP Among Clients at Sex Worker and MSM Facilities in South Africa,” *PloSOne* (2020), https://doi.org/10.1371/journal.pone.0228620.

53 Chi Mgbako, Katherine Glenn Bass, Erin Bundra, Mehak Jamil, Jere Keys, and Lauren Melkus, “The Case for Decriminalising Sex Work in South Africa,” *Georgetown Journal of International Law* 44 (2017): 1423-1454.

54 Kelly Kilburn, Meghna Ranganathan, Marie C.D. Stoner, James P. Hughes, Catherine MacPhail, Yaw Agyei, F. Xavier Gómez-Olivé, Kathleen Kahn, Audrey Pettifor, “Transactional Sex and Incident HIV Infection in a Cohort of Young Women from Rural South Africa,” *AIDS* 32 (2018): 1669-1677.

55 Hayley MacGregor, “Mapping the Body: Tracing the Personal and Political Dimensions of HIV/AIDS in Khayelitsha,” *Anthropology and Medicine* 16 (2007): 85-95.

56 Rebecca Hodes, *Broadcasting the Pandemic: A History of HIV on South African Television* (Cape Town: HSRC Press, 2014).

57 Mbali, *South African AIDS Activism and Global Health Politics.*

58 Kaushaik Sunder Rajan, *Biocapital: The Constitution of Postgenomic Life* (Durham:Duke University Press, 2006), 3.

59 Mandisa Mbali, “From AIDS to Cancer: Health Activism, Biotechnology and Intellectual Property in South Africa,” *Social Dynamics* 46 (2020): 449-470. For deeper exploration of how we can historicise cancer and associated industries (including the pharmaceutical industry), see S. Lochlann Jain, *Malignant: How Cancer Becomes Us* (Berkeley: University of California Press, 2013). The author uses the idea of cancer “becoming us [American]” through two mechanisms: firstly, cancer is part of American history; secondly it exists at a micro- (bodily) and macro- (institutional and society-wide) level. By contrast, when I discuss the notion of viruses “becoming us” in the conclusion, I am referring to interaction between different levels of life in relation to a global health emergency triggered by an infectious disease. I also depart from the author’s autoethnographic approach as a cancer patient.

60 Petar Jandrić, “Bioinformationalism: Information theory and genomics,” in Peters, Jandrić, and McLaren, “Viral modernity?”

61 Ibid. Here Jandrić cites Craig Venter, who is controversial in his own right due to his efforts to patent the Human Genome at the beginning of this century.

62 Ibid., 7.

63 Ibid.

64 Ibid.

65 K. Crowe, “‘We’re opening everything’: Scientists share coronavirus data in unprecedented way to contain, treat disease,” *CBC News*, 1 February 2020, https://www.cbc.ca/news/health/coronavirus-2019-ncov-science-virus-genome-who-research-collaboration-1.5446948.

66 Ibid.

67 Jeffrey Barrett, “Scientists Sharing Omicron Data Were Heroic. Let’s Ensure They Don’t Regret It,” *The Guardian*, 28 November 2021, https://www.theguardian.com/commentisfree/2021/nov/28/scientists-sharing-omicron-date-were-heroic-lets-ensure-they-dont-regret-it.

68 Keymantri Moodley, Nezerith Cengiz, Aneeka Domingo, Gonasagrie Nair, Adetayo Emmanuel Obasa, Richard John Lessells, Tulio de Oliveira, “Ethics and Governance Challenges Related to Genomic Data Sharing in Southern Africa: The Case of SARS-CoV-2,” *Lancet: Global Health* 10 (2022): E1855-E1859.

69 Ibid.

70 Mbali*, South African AIDS Activism and Global Health Politics.*

71 Ellen t’Hoen, *The Global Politics of Pharmaceutical Monopoly Power* (Diemen: AMB Publishers, 2009).

72 Olivia Goldhill, Rosa Furneaux, and Malden Davies, “’Naively Ambitious’: How COVAX Failed on Its Promise to Vaccinate the World,” *Statnews*, 8 October 2021, https://www.statnews.com/2021/10/08/how-covax-failed-on-its-promise-to-vaccinate-the-world/.

73 For more on vaccine “hoarding” see “Access to COVID-19 Vaccines: Looking Beyond COVAX,” *Lancet* 397 (2021): 941.

74 Goldhill, Furneaux, and Davies, “‘Naively Ambitious.’”

75 Ibid.

